# Role of Neuropilin-1/Semaphorin-3A signaling in the functional and morphological integrity of the cochlea

**DOI:** 10.1371/journal.pgen.1007048

**Published:** 2017-10-23

**Authors:** Pezhman Salehi, Marshall X. Ge, Usha Gundimeda, Leah Michelle Baum, Homero Lael Cantu, Joel Lavinsky, Litao Tao, Anthony Myint, Charlene Cruz, Juemei Wang, Angeliki Maria Nikolakopoulou, Carolina Abdala, Matthew William Kelley, Takahiro Ohyama, Thomas Matthew Coate, Rick A. Friedman

**Affiliations:** 1 USC-Tina and Rick Caruso Department of Otolaryngology-Head & Neck Surgery, Zilkha Neurogenetic Institute, USC Keck School of Medicine, University of Southern California, Los Angeles, California, United States of America; 2 Department of Anatomy and Neurobiology, Northeast Ohio Medical University, Rootstown, Ohio, United States of America; 3 Department of Medicine, Temple University School of Medicine, Philadelphia, Pennsylvania, United States of America; 4 Department of Biology, Georgetown University, Washington, D.C., United States of America; 5 Graduate Program in Surgical Sciences, Federal University of Rio Grande do Sul, Porto Alegre, Rio Grande do Sul, Brazil; 6 Stem Cell Biology & Regenerative Medicine, Keck School of Medicine, University of Southern California, Los Angeles, California, United States of America; 7 Department of Physiology and Biophysics, Zilkha Neurogenetic Institute, USC Keck School of Medicine, University of Southern California, Los Angeles, California, United States of America; 8 National Institute on Deafness and Other Communication Disorders, Bethesda, Maryland, United States of America; University of California San Diego, UNITED STATES

## Abstract

*Neuropilin-1 (Nrp1)* encodes the transmembrane cellular receptor neuropilin-1, which is associated with cardiovascular and neuronal development and was within the peak SNP interval on chromosome 8 in our prior GWAS study on age-related hearing loss (ARHL) in mice. In this study, we generated and characterized an inner ear-specific *Nrp1* conditional knockout (CKO) mouse line because *Nrp1* constitutive knockouts are embryonic lethal. *In situ* hybridization demonstrated weak *Nrp1* mRNA expression late in embryonic cochlear development, but increased expression in early postnatal stages when cochlear hair cell innervation patterns have been shown to mature. At postnatal day 5, *Nrp1* CKO mice showed disorganized outer spiral bundles and enlarged microvessels of the stria vascularis (SV) but normal spiral ganglion cell (SGN) density and presynaptic ribbon body counts; however, we observed enlarged SV microvessels, reduced SGN density, and a reduction of presynaptic ribbons in the outer hair cell region of 4-month-old *Nrp1* CKO mice. In addition, we demonstrated elevated hearing thresholds of the 2-month-old and 4-month-old *Nrp1* CKO mice at frequencies ranging from 4 to 32kHz when compared to 2-month-old mice. These data suggest that conditional loss of *Nrp1* in the inner ear leads to progressive hearing loss in mice. We also demonstrated that mice with a truncated variant of *Nrp1* show cochlear axon guidance defects and that exogenous semaphorin-3A, a known neuropilin-1 receptor agonist, repels SGN axons *in vitro*. These data suggest that Neuropilin-1/Semaphorin-3A signaling may also serve a role in neuronal pathfinding in the developing cochlea. In summary, our results here support a model whereby Neuropilin-1/Semaphorin-3A signaling is critical for the functional and morphological integrity of the cochlea and that *Nrp1* may play a role in ARHL.

## Author summary

Neuropilin-1 is a member of the neuropilin family acting as an essential cell surface receptor involved in semaphorin-dependent axon guidance and VEGF-dependent angiogenesis and lies within our previously identified ARHL GWAS interval. In this study, we investigated the role of Neuropilin-1/Semaphorin-3A signaling in the functional and morphological integrity of the cochlea, specifically the innervation and vascularization patterns. Detailed analyses of the cochleae of 4-month-old *Nrp1* CKO mice showed abnormalities in ribbon synapses, innervation of the hair cells, and microvessels of the stria vascularis. We show also that Neuropilin-1/Semaphorin-3A signaling plays an important role in cochlear innervation.

## Introduction

Age-related hearing loss (ARHL), or presbycusis, is a progressive bilateral symmetrical sensorineural hearing loss [1] characterized by four types of pathology: (1) sensory deficits resulting from loss of outer hair cells as seen in loss of high frequency auditory brainstem response, (2) neural deficits from auditory nerve degeneration resulting in poor speech recognition, (3) degeneration of the stria vascularis leading to flat audiometric losses across frequencies; and (4) cochlear conductive deficits associated with increased stiffness of the basilar membrane resulting in evenly sloping audiometric losses [2]. Familial studies of presbycusis have attributed approximately half of audiometric variances to hereditary factors; however, the highly variable age of onset, disease progression, and severity of ARHL demonstrate the current uncertain contribution of individual genetic factors to cochlear integrity [3]. Our group has recently demonstrated that ARHL in humans is a polygenic trait [4]. Human genetic studies suggest associations between ARHL and several genes including GRHL2, ITGA8, IQGAP2, GRM7, PCDH15, PCDH20, APOE, EDN1, ESRRG [2]. Although very little is known about ARHL in humans, numerous studies have been published on ARHL in mice. A genetic component to ARHL in inbred mice has been described with approximately 18 Mendelian loci reported to date [5–8]. It has been our overriding hypothesis that true ARHL in mice, as in humans, is a polygenic trait with the composite phenotype resulting from genomic variation at multiple loci likely different from the Mendelian loci described thus far.

To define the genetic architecture of ARHL in mice, we undertook a genome-wide association study (GWAS) using a meta-analysis strategy by combining data sets from five cohorts containing 937 samples in total [9]. The results of the meta-analysis led us to an approximately 2 Mb interval containing *Nrp1*, a gene that is involved in cardiovascular and neuronal development and is closely related to *Neuropilin-2* (*Nrp2*), a gene involved in cochlear epithelial innervation [10]. Neuropilin-1 is a transmembrane receptor type I protein that is known to bind both vascular endothelial growth factor beta (VEGFb) and semaphorin classes including subtypes 3A, 3B, 3C, and 3D. Semaphorin-3A is involved in axonal guidance via chemorepulsion [11]. Semaphorin-3A -induced neuronal growth cone collapse has been shown to require neuropilin-1 in conjunction with Plexin-A co-receptors [12]. Previous cardiovascular studies have shown that altered endothelial cell migration, abnormal blood flow, and enlarged vessels are major defects caused by targeted inactivation of the *Nrp1* gene in mice. Additionally, homozygous *Nrp1* mutant mice are known to have a perinatal lethal phenotype due to impaired heart development [13].

In this study, using an inner ear-specific knock out, we investigated the role of *Nrp1* in the functional and morphological integrity of the cochlea in mice. The results of this study suggest *Nrp1* may be involved in ARHL.

## Results

### The complementary spatial and temporal expression patterns of *Nrp1* and *Sema3a* support their roles in refinement of cochlear innervation in the immediate postnatal period

**Fig 1:**
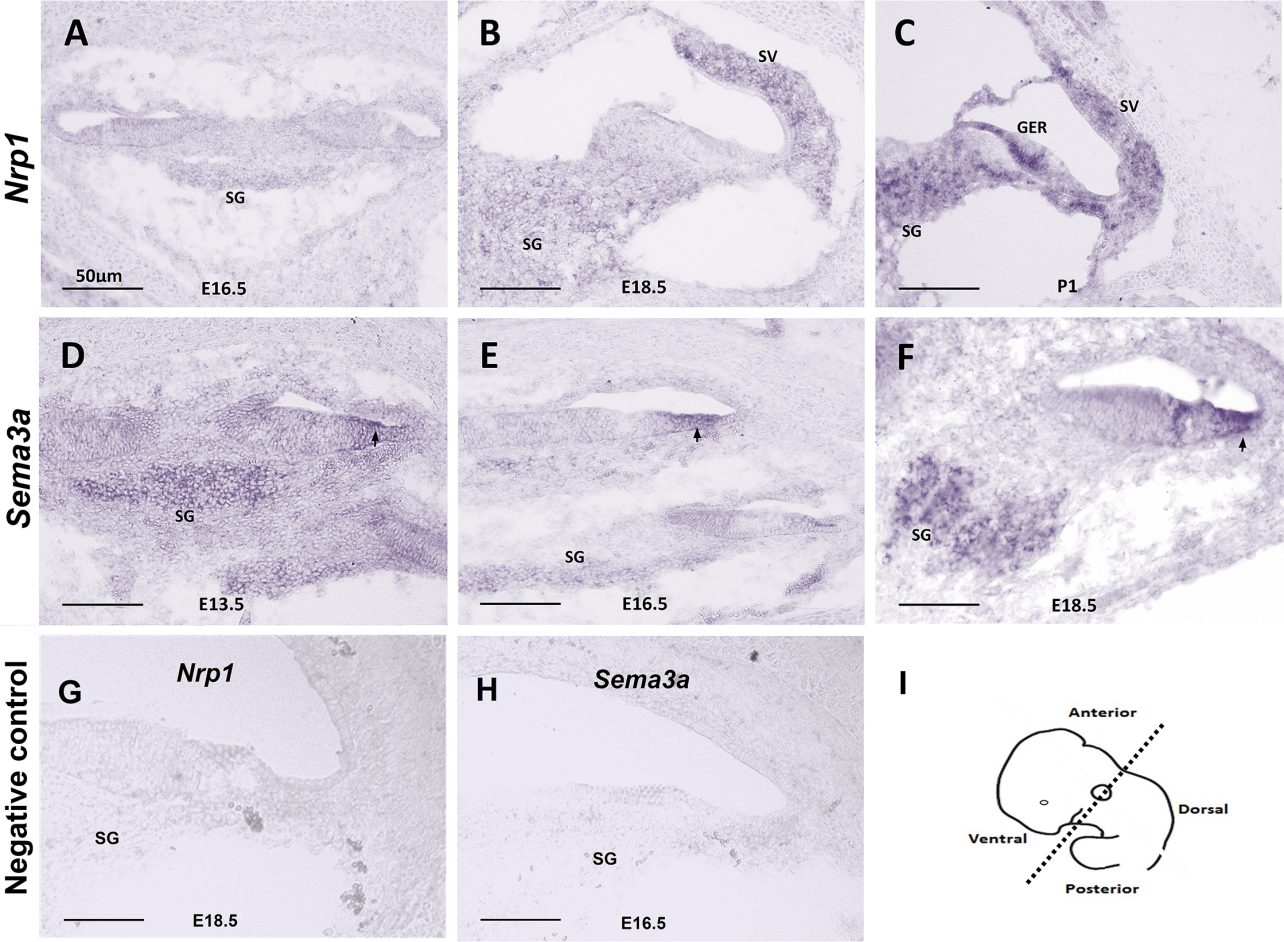
*In situ* hybridization of *Nrp1* and *Sema3a*. *In situ* hybridization showing the expression pattern of *Nrp1* (A-C) and *Sema3a* (D-F) in mice at time points E13.5, E16.5, E18.5, and P1. *Nrp1* was not expressed at E16.5 (A), weakly expressed in the SG and SV at E18.5 (B), and more highly expressed in the SG, greater epithelial region (GER), and SV at P1 (C). *Sema3a* expression pointed by arrows on the abneural side of the cochlear epithelium was observed around E13.5 (D) and continued at E16.5 (E) and E18.5 (F) on the same region of the cochlear epithelium and spiral ganglion neurons. (G, H) Negative controls for *Nrp1* (E18.5) and *Sema3a* (E16.5) using sense probes. (I) Panel I shows the plane of sections. SG: spiral ganglion. SV: stria vascularis. Scale bar = 50 μm.

We first used *in situ* hybridization to characterize the expression of *Nrp1* and its ligand *Sema3a* at different stages of cochlear development. Between E13.5 and P1, the SGNs migrate along the extending cochlear duct then extend peripheral axons toward the cochlear epithelium and central axons toward the brainstem [14]. *In situ* hybridization of WT cochleae at E13.5, E15.5, E18.5, and P1 showed weak expression of *Nrp1* at E16.5 and E18.5 with more robust expression starting at P1 (Fig 1A and 1C). Our *in situ* hybridization data also showed that *Sema3a* expression started around E13.5 and continued at E16.5 and E18.5 on the abneural side of the cochlear epithelium and SGNs (Fig 1D–1F). These data suggest that both *Nrp1* and *Sema3a* are expressed in the cochlea during time points when SGNs begin to innervate the organ of Corti.

**Fig 2:**
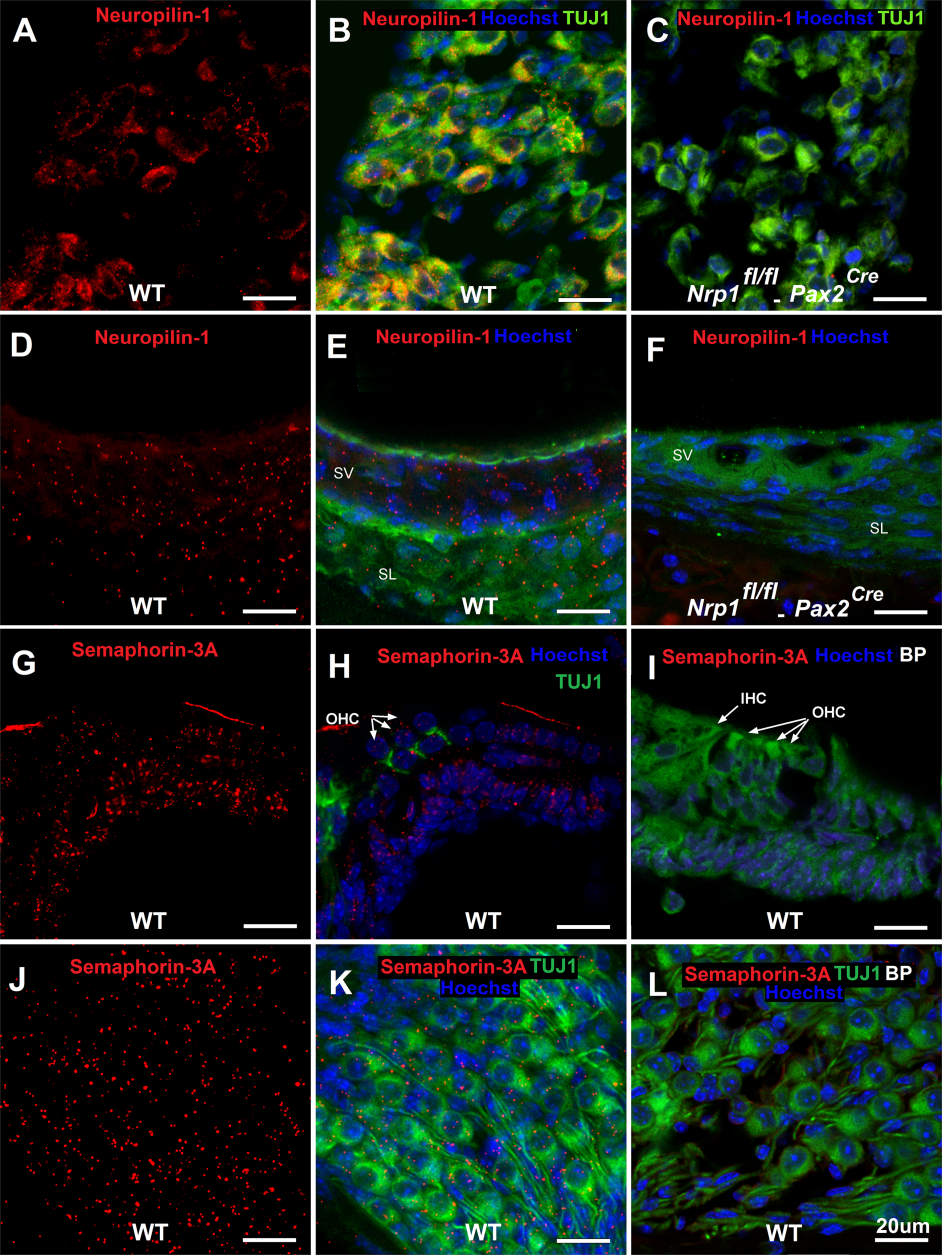
Immunostaining of neuropilin-1 in the SGNs/SV and of Sema3a in the organ of Corti and SGNs in mice at postnatal stage (P5). Mid-modiolar sections of the cochlea were immunostained with antibodies to TUJ1, neuropilin-1, and Sem3a. Blocking peptide (BP) for semaphorin-3A was used as a negative control. Neuropilin-1 receptors were expressed on SGNs and SV in WT mice at P5 (A, B, D, E) and were not expressed on SGNs (C) and SV (F) of *Nrp1*^*fl/fl*^;*Pax2*^*Cre*^ mice. Semaphorin-3A expression was detected on organ of Corti (G, H), and SGNs (J, K) at postnatal day 5 (D, E). Panel I and L show semaphorin-3A immunostaining of the organ of Corti (I) and SGNs (L) in presence of blocking peptide for semaphorin-3A. Green in panel E, F, I is background color from TUJ1 staining. Scale bar = 20 μm.

We next performed immunostaining using P5 cochleae to determine the precise distribution of neuropilin-1 and semaphorin-3A in the postnatal cochlea. As shown in Fig 2, neuropilin-1 is visible in SGNs and the SV (Fig 2A, 2B, 2D and 2E), but not expressed after conditional deletion of *Nrp1* (Fig 2C and 2F). Semaphorin-3A protein was visible within the organ of Corti (Fig 2G and 2H) and SGNs (Fig 2J and 2K), but not after the semaphorin-3A antibody was pre-adsorbed by the blocking peptide (Fig 2I and 2L). Overall, these data suggest that *Nrp1* is expressed at minimal levels by SGNs and cells of the stria vascularis before birth while *Nrp1* levels become elevated in these locations after birth. *Sema3a* is expressed by cells within the cochlear epithelium and SGNs, which is complementary to the expression patterns of *Nrp1*. The simultaneous expression of both neuropilin-1 and semaphorin-3A shortly after birth suggested these factors may be involved in the process of SGN pruning and refinement, which occurs during this time in cochlear development. These findings prompted further investigation of Neuropilin-1/Sema-3A signaling in cochlear innervation.

Much of the genetic data from our meta-analysis GWAS came from the original backcrossing data (C57BL/6J x DBA/2J) during the mapping of *Ahl8* [6]. In their mapping study of ahl8, a locus on chromosome 8 was also identified. This led us to determine the possibility of *Nrp1* expression variation in the cochlear tissue of C57BL/6J and DBA/2J mice. Real-time PCR showed 1.78-fold higher *Nrp1* expression for DBA/2J mice (1.96) when compared to C57BL/6J (1.09) (S1 Fig) supporting our gene selection (p<0.01).

### The number of OHC ribbon synapses and SGN density are reduced in 4-month-old *Nrp1* CKO mutants

**Fig 3:**
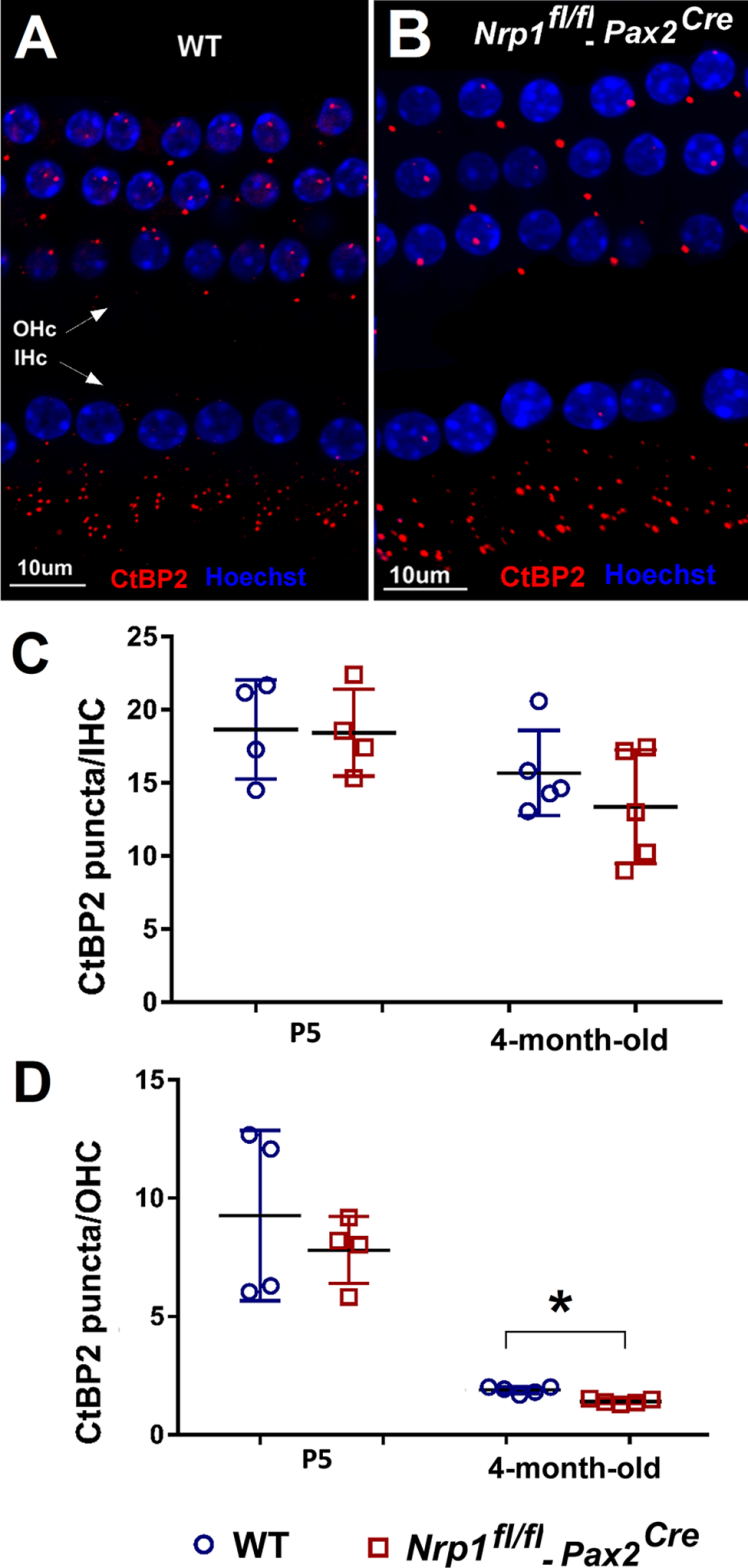
Presynaptic marker CtBP2 counts of the IHCs and OHCs. Cochlear whole mounts of WT, *Nrp1*^*fl/fl*^;*Pax2*^*Cre*^ mice at P5 (n = 4 per group) and 4 months (n = 5 per group) were immunostained with presynaptic marker CtBP2. Panel A and B shows CtBP2 immunostaining of the 4-month-old mice (A, B). The number of CtBP2 puncta was compared separately to the number of inner and outer hair cells per section. CtBP2 ratios of IHCs did not differ significantly between WT and *Nrp1*^*fl/fl*^;*Pax2*^*Cre*^ mutants from P5 to maturity (C). OHC synaptic ribbon ratios of 4-month-old *Nrp1*^*fl/fl*^;*Pax2*^*Cre*^ demonstrated a significant decrease in CtBP2 counts in the basal turn (p<0.05). CtBP2 counts of the IHCs showed two cochlea out of five with nearly 50% decreased numbers of the puncta when compared to WT controls; however, the average of the counts (five cochlea) did not show a statistically significant difference (D). *p<0.05. Scale bar = 10 μm.

**Fig 4:**
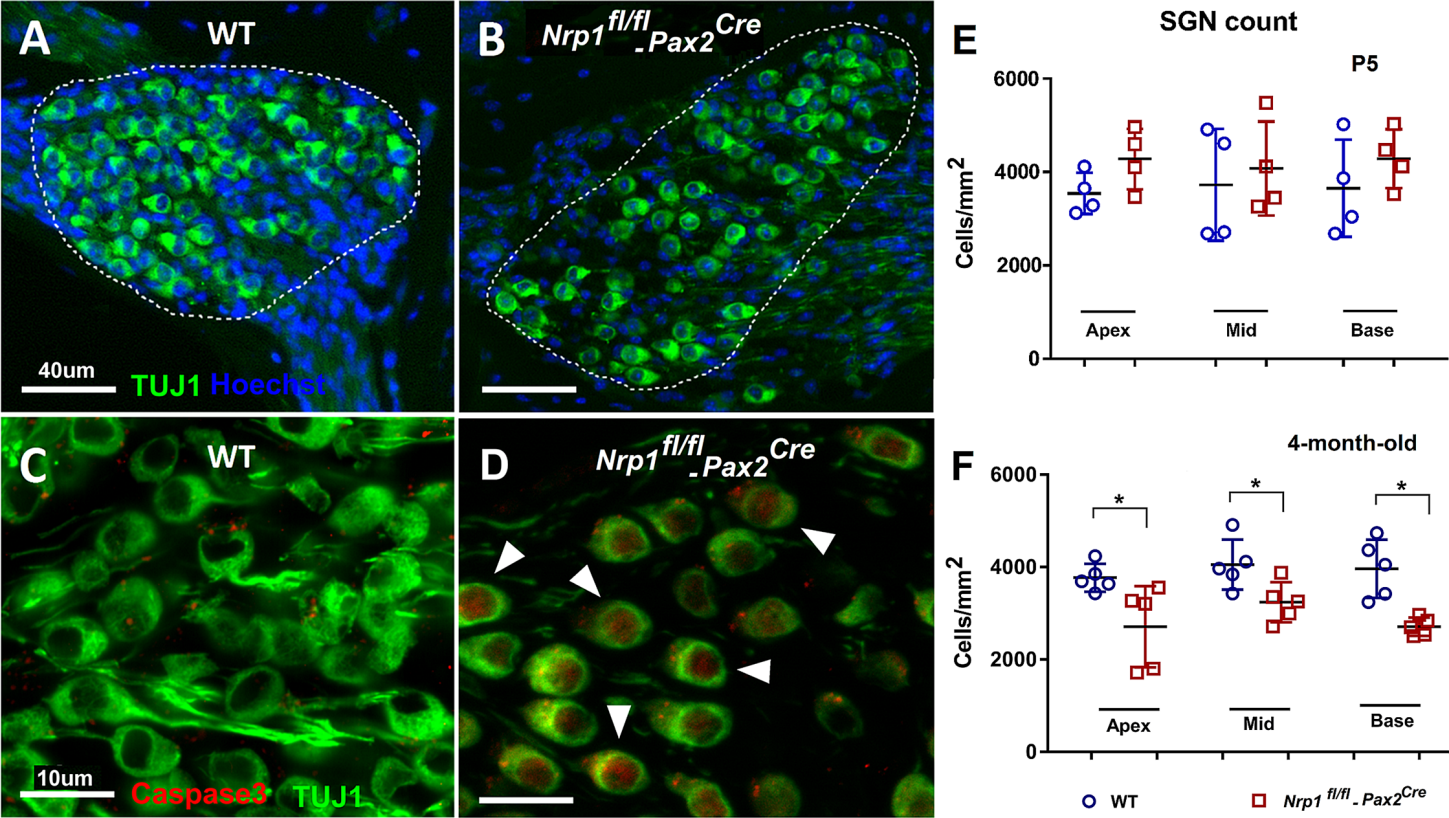
SGN density and caspase-3 expression in 4-month-old WT and *Nrp1*^*fl/fl*^;*Pax2*^*Cre*^ mutant mice. Mid-modiolar sections of the cochlea (basal turn) showing lower density of the SGNs in 4-month-old *Nrp1*^*fl/fl*^;*Pax2*^*Cre*^ mice compared to WT controls (A, B). Cochlear SGN of 4-month-old WT and *Nrp1*^*fl/fl*^;*Pax2*^*Cre*^ mice were immunostained with antibodies to Caspase-3 (Red) to ascertain the fate of the SGN at cochlear maturity (C, D). Caspase-3 positive SGNs pointed by arrows in panel D (red staining) showing apoptosis of neurons in 4-month-old *Nrp1*^*fl/fl*^;*Pax2*^*Cre*^ mice compared to WT controls on panel C. (E) There was no statistically significant difference between SGNs count of the *Nrp1*^*fl/fl*^;*Pax2*^*Cre*^ mice at P5 when compared to WT controls (n = 4 per group). SGNs count at the apical, middle, and basal (n = 5 per group) turns of the cochlea was consistently lower in the 4-month-old *Nrp1*^*fl/fl*^;*Pax2*^*Cre*^ mutant group compared to WT controls (F). *p<0.05. Scale bar = 40 μm for A, and B. Scale bar = 10 μm for C, and D.

To investigate the function of neuropilin-1 in the cochlea, we generated an inner-ear specific conditional knockout mouse using *Pax2*^*Cre*^ and loxp-driven *Nrp1* removal (see Methods for details). Using this line, we first wanted to determine whether *Nrp1* is required for the formation or maintenance of ribbon bodies, which represent glutamatergic synapses connecting hair cells and SGNs. To visualize and quantify ribbon bodies, cochlear whole mount preparations from WT, *Nrp1*^*fl/+*^*;Pax2*^Cre^ and *Nrp1*^*fl/fl*^*;Pax2*^Cre^ mice at P5 (n = 4 per group) and 4 months (n = 5 per group) were immunostained with antibodies that bind to ribeye, a splice variant of CtBP2. The tissue samples were stained with Hoechst33342 to confirm whether the ribbon bodies were located on either OHCs or IHCs. The time points described above were chosen so that we could track any possible changes in synaptic connectivity from just after birth to full maturity within the cochlea. CtBP2 counts of both IHCs and OHCs at P5 showed no differences between WT and *Nrp1* CKO samples (Fig 3). No significant changes in immunostaining of IHCs was observed between *Nrp1*^*fl/fl*^*;Pax2*^Cre^ and WT at 4 months; however, at this time point, the synaptic ribbon density (CtBP2 counts) in the OHC region in 4-month-old mice was significantly reduced (p<0.01) for *Nrp1*^*fl/fl*^*;Pax2*^Cre^ mutants (1.9 puncta/cell) compared to the controls (1.4 puncta/cell). Given this reduction in ribbon synapses, we next wanted to determine whether *Nrp1* loss also conferred a loss of SGNs (possibly through apoptosis). Thus, we quantified SGN density at the apical, middle, and basal turns of the cochlea at P5 (n = 4 per group) and at 4-months (n = 5 per group) for WT, *Nrp1*^*fl/+*^*;Pax2*^Cre^ and *Nrp1*^*fl/fl*^*;Pax2*^Cre^ mice (Fig 4). As expected from our ribbon synapse counts, at P5 no significant changes in SGN density were observed among the different genotypes or regions of the cochlea (Fig 4E). However, the SGN counts in 4-month-old mice (Fig 4F) showed decreased density of the neuronal cell bodies in *Nrp1*^*fl/fl*^*;Pax2*^Cre^ mice compared to WT controls (apical turn p = 0.03, middle turn p = 0.03, and basal turn p = 0.002). Interestingly, the number of SGNs lost in the absence of *Nrp1* is unexpectedly high compared to the number of IHC ribbon synapses lost (see Fig 3 and Discussion). Nevertheless, the loss of OHC synaptic ribbons and diminished SGN density in 4-month-old *Nrp1*^*fl/fl*^*;Pax2*^Cre^ mice, suggests that *Nrp1* may play a role in maintaining SGN integrity during postnatal stages.

To examine the mechanism of SGN cell loss, we performed caspase-3 immunostaining. Caspase-3, a molecule necessary for the cellular apoptotic cascade, was identified by immunostaining in WT and *Nrp1*^*fl/fl*^*;Pax2*^Cre^ cochleae to ascertain the fate of the SGNs once mice reached 4 months of age. Caspase-3 positive neurons were found in *Nrp1*^*fl/fl*^*;Pax2*^Cre^ mutants but not in WT mice, suggesting that the loss of OHC ribbon synapses resulted from pruning or the apoptosis of mature neurons (Fig 4C and 4D). Taken together, these data suggest a gradual loss of contacts between OHCs and SGNs in the absence of *Nrp1*, which correlates with the death of SGNs around 4 months of age.

### *Nrp1* CKO shows abnormal OHC innervation

**Fig 5:**
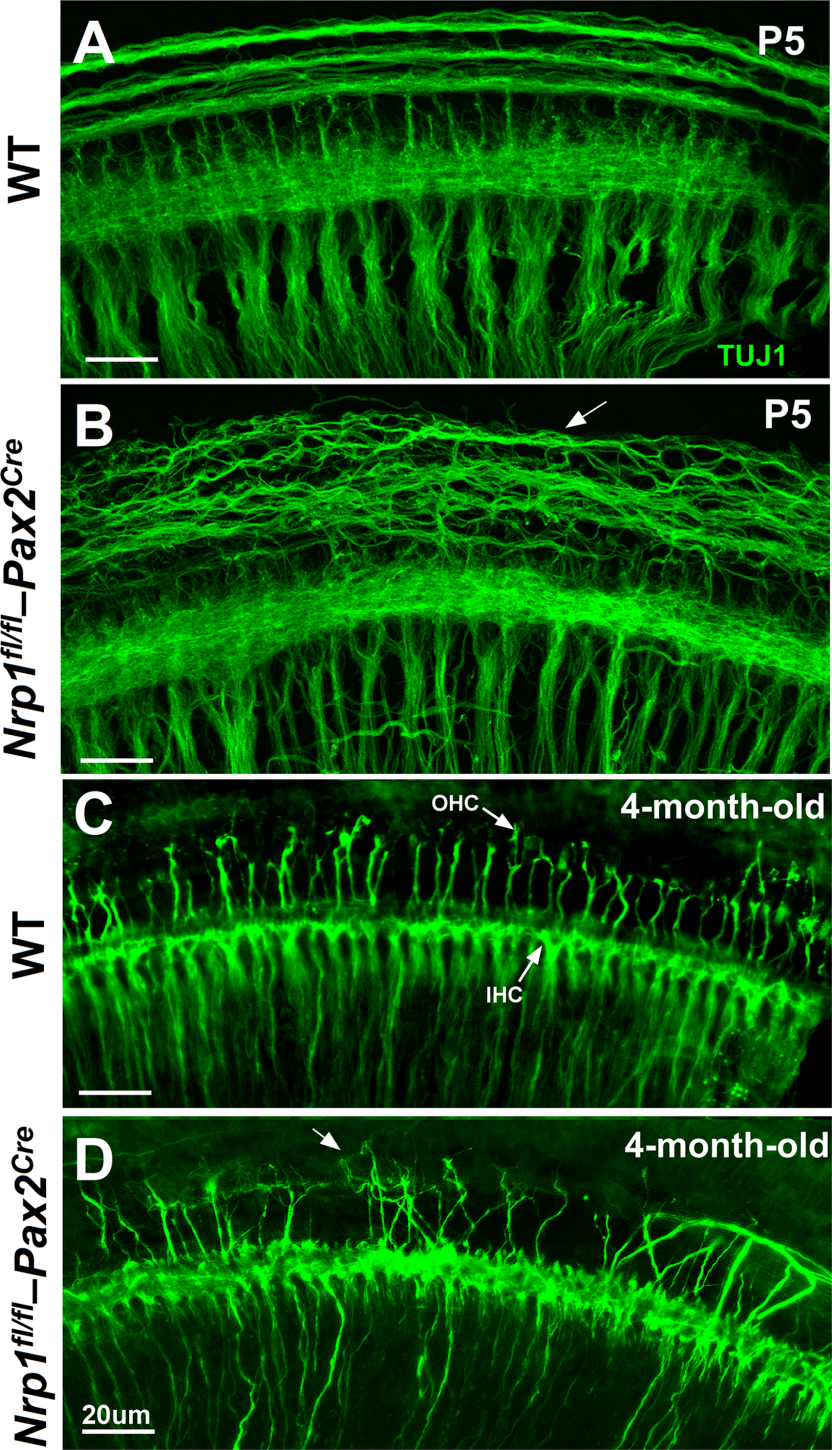
SGN projections in the basal turns of cochleae of WT and *Nrp1*^*fl/fl*^;*Pax2*^*Cre*^ mice at P5 and 4-month-old age. Disorganized outer spiral bundles were evident in cochleae of the *Nrp1*^*fl/fl*^;*Pax2*^*Cre*^ mice (P5) in the basal turn (n = 3 per group). The arrow shows disorganized outer spiral bundles (B). TUJ1 immunostaining of the SGN fibers extending into the inner and outer hair cell regions in 4-month-old *Nrp1*^*fl/fl*^;*Pax2*^*Cre*^ mutants revealed aberrant axons with abnormal innervation of the OHCs. Arrow shows abnormal innervation of the OHCs (D). WT mice did not show aberrant axon migration or innervation. Scale bar = 20 μm.

**Fig 6:**
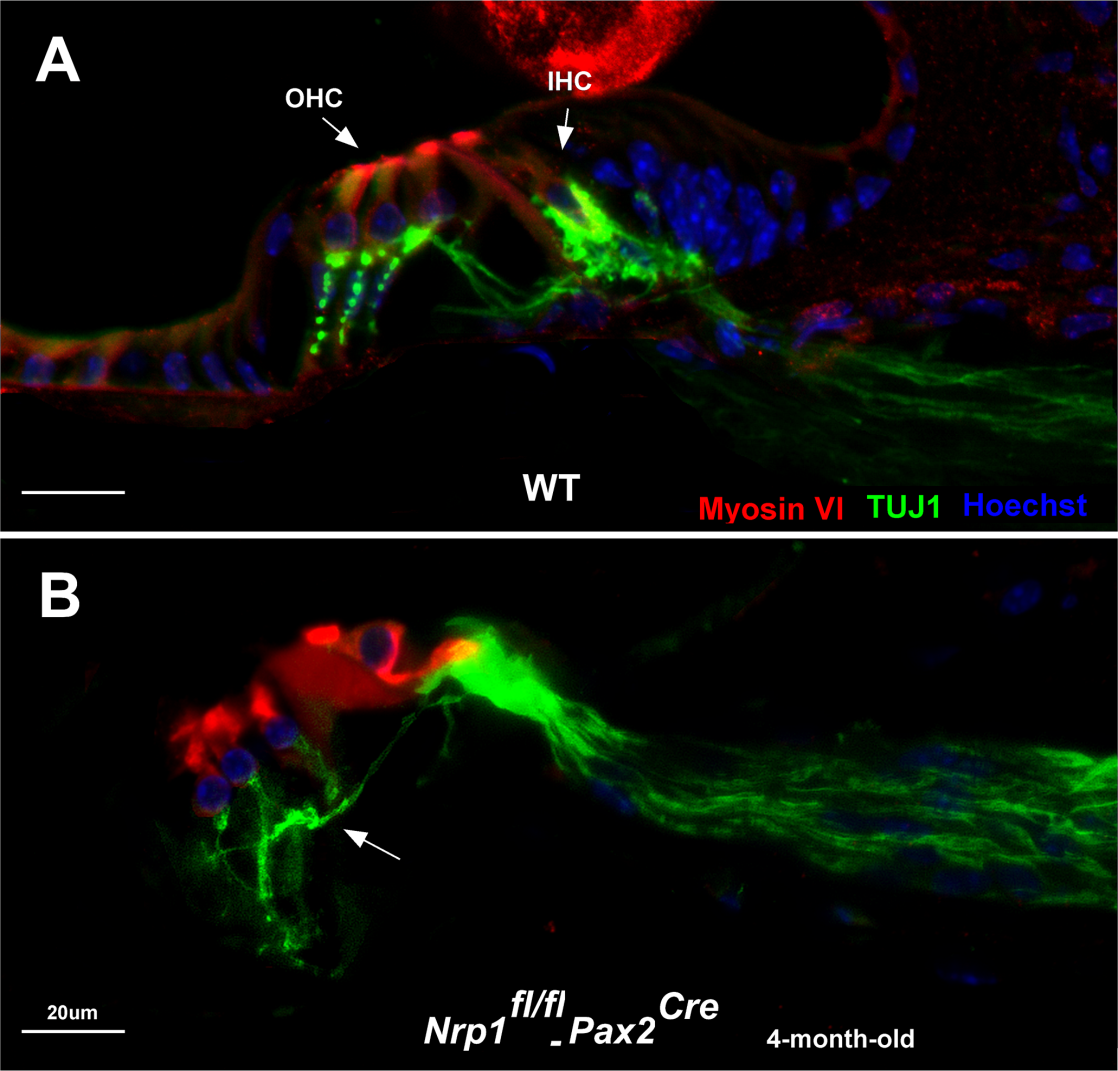
Aberrant innervation of OHCs in *Nrp1*^*fl/fl*^;*Pax2*^*Cre*^ mice at 4 months of age. TUJ1 immunostaining of the mid-modiolar cross-section of cochleae of 4-month-old WT (A) and *Nrp1*^*fl/fl*^;*Pax2*^*Cre*^ mice reveals disorganized innervation of the OHCs in *Nrp1-*CKO compared to WT controls. Arrow shows abnormal innervation of the OHCs (B). Scale bar = 20 μm.

We next wanted to see if *Nrp1*^*fl/fl*^*;Pax2*^Cre^ mutants also showed defects in cochlear innervation patterns to determine the extent to which Nrp1 may function in axon guidance in the cochlea. Cochleae from WT and *Nrp1*^*fl/fl*^*;Pax2*^Cre^ mutants at P5 and 4 months were immunostained with TUJ1 antibodies and assessed as whole-mount preparations. At P5, disorganized outer spiral bundles (type II fibers) were evident in cochleae of the *Nrp1*^*fl/fl*^*;Pax2*^Cre^ mice at basal, mid, and apical turns (n = 3 per group), but the radial fibers (mostly type I SGNs) appeared normal (Fig 5A and 5B). Compared to controls, we also observed significant disruptions to the normal patterns of innervation in cochleae from 4-month-old *Nrp1*^*fl/fl*^*;Pax2*^Cre^ mice (Fig 5C and 5D). In a normal cochleae, 90–95% of the SGNs innervate IHCs; the remaining 5–10% of neurons travel beyond IHCs to innervate OHCs in an *en passant* fashion [15]. TUJ1 immunostaining of cochlear nerve fibers extending into the hair cell region in 4-month-old *Nrp1*^*fl/fl*^*;Pax2*^Cre^ mutants (basal turn) revealed aberrant axons with abnormal innervation of OHCs. Mid-modiolar cross-sections of the cochlea of the *Nrp1*^*fl/fl*^*;Pax2*^Cre^ mice (4-month-old) also showed disorganized innervation of the outer hair cells (Fig 6).

### *Nrp1*^*sema-/sema-*^ cochleae show cochlear innervation defects that are similar to the *Nrp1* CKO

**Fig 7:**
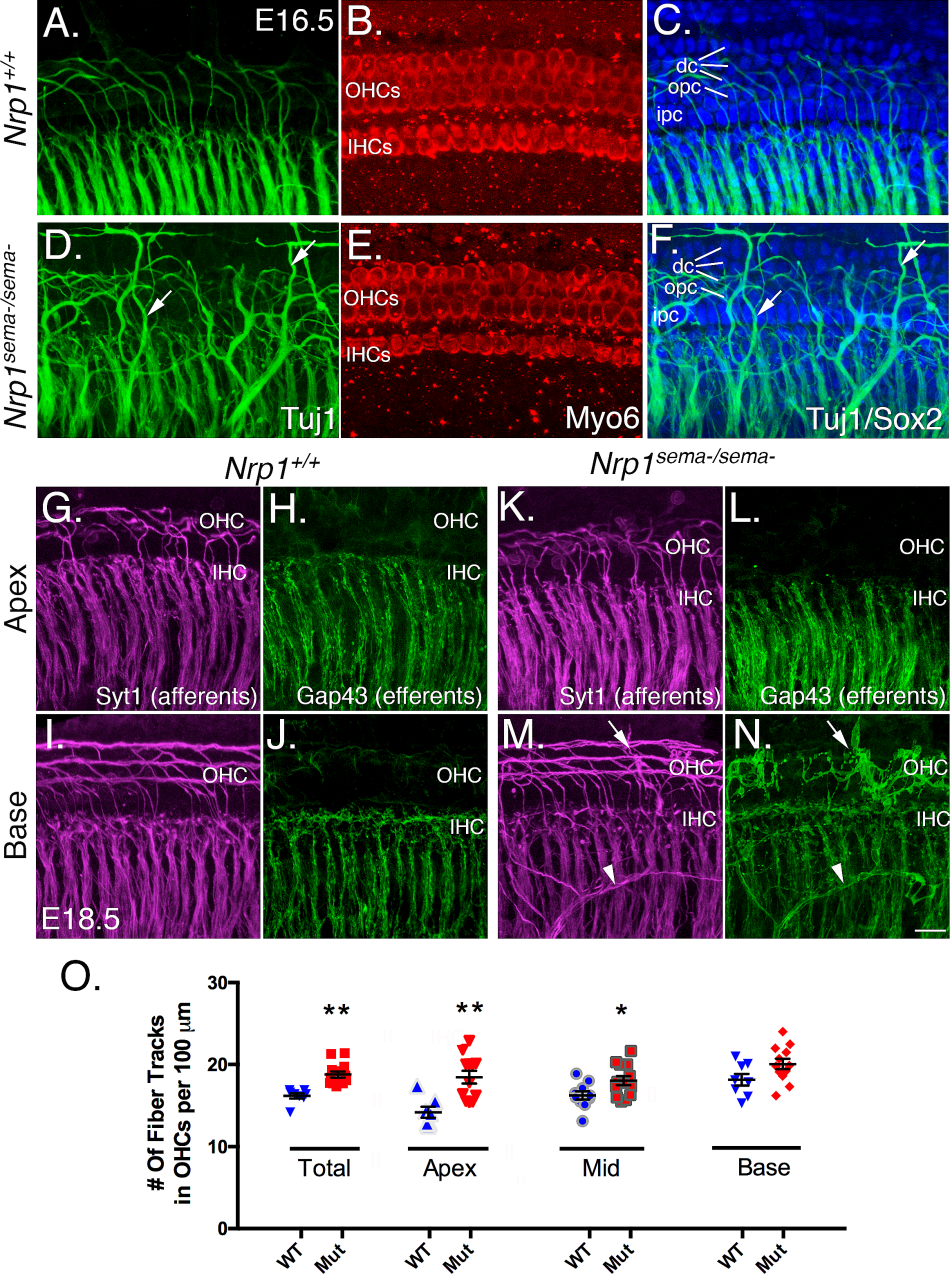
Loss of sema-mediated activation of neuropilin-1 causes increased sensory domain innervation. (A-F) Whole mount samples from E16.5 cochleae from WT and *Nrp1*^*sema-/sema-*^ mice. Neurons were stained with anti-TUJ1 antibodies (green) and hair cells were stained with anti-Myo6 antibodies (red). The arrows in D and F point to excessive nerve fibers innervating the hair cell region. Panels C and D show TUJ1 labeling with Sox2 immunolabeling, which identifies the different supporting cell types. OHC, outer hair cell; IHC, inner hair cell; dc, Deiters’ cell; opc, outer pillar cell; ipc, inner pillar cell. (G-N) Analysis of SGN afferents (Syt1 staining; magenta) and olivocochlear efferents (Gap43 staining; green) from E18.5 cochleae from WT and *Nrp1*^*sema-/sema-*^ mice. The arrows in panels M and N point to an aberrant nerve fiber in the OHC region of *Nrp1*^*sema-/sema-*^ cochleae. The arrowheads in M and N point to a cluster of nerve fibers that appears to be traversing across the cochlear radial fibers in an unusual manner. (O) Panel O showing that loss of Sema-mediated activation of neuropilin-1 can lead to a significant increase in Syt+ SGN processes projecting into the OHC region.

Neuropilin-1 can be activated by both secreted semaphorins and Vascular Endothelial Growth Factors (VEGFs) [16]. Given this, we wanted to ask next whether mice with a variant of *Nrp1* that fails to bind secreted semaphorins (*Nrp1*^*sema-*^) showed cochlear innervation defects similar to the *Nrp1* CKO line [17]. We first used anti-TUJ1 antibodies to examine the overall distribution of nerve fibers in mutant and WT cochleae at E16.5 (Fig 7A–7F). For each sample, we also performed anti-Myo6 and anti-Sox2 immunostaining to identify the hair cells and supporting cells, respectively. Compared to cochleae from WT littermates (Fig 7A–7C), cochleae from *Nrp1*^*sema-/sema-*^ mice showed nerve fibers in great excess with many that extended past the OHC region and even sometimes past the Deiters’ cell region (Fig 7D–7F). Cochleae from *Nrp1*^*sema-/sema-*^ mice showed a normal distribution of hair cells and supporting cells (Fig 7B, 7C, 7E and 7F) indicating the innervation defects here were not due to changes in organ of Corti formation. Using E18.5 samples from the *Nrp1*^*sema-/sema-*^ mice, we next delineated the distribution of SGN afferents using Syt1 antibodies and cochlear efferents using Gap43 antibodies [10]. Compared to the apex and middle regions of control cochleae, *Nrp1*^*sema-/sema-*^ cochleae showed a significant increase in Syt+ fibers in the OHC region (Fig 7K and 7O), but no changes in the distribution of Gap43+ efferent fibers. At the base, we found no significant increases in Syt+ fibers in the OHC region of *Nrp1*^*sema-/sema-*^ cochleae overall (Fig 7) but did often see unusual nerve bundles that were both Syt1- and Gap43-positive (see arrowheads in 7M and N). These unusual bundles often took torturous paths toward the organ of Corti and terminated in the hair cell region or just beyond. In addition, the anatomical origins of these neurons were not clear in that the processes appeared to come from outside of the cochlea and not Rosenthal’s canal where the SGN cell bodies are located. Nevertheless, cochleae from *Nrp1*^*sema-/sema-*^ mice showed innervation defects that, to a large extent, mirrored the phenotypic defects in the *Nrp1* CKO mice. This indicates that neuropilin-1 receptor activation by secreted semaphorins is necessary for normal cochlear innervation.

### siRNA mediated knockdown of *Nrp1* decreases semaphorin-3A action

**Fig 8:**
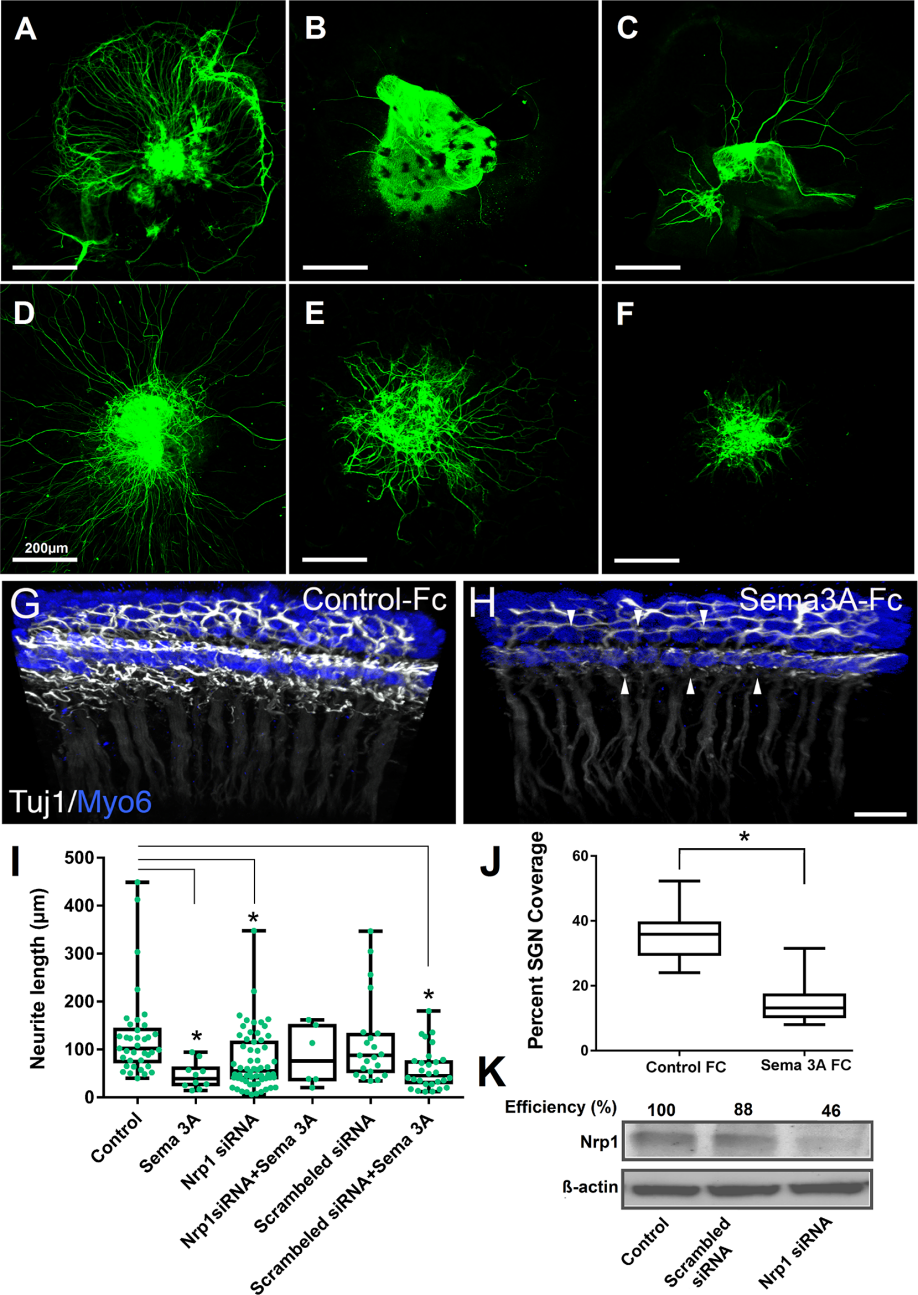
*Nrp 1* mediates the semaphorin-3A-induced inhibition of neurite outgrowth. SGN explants were transfected with either 50nM *Nrp1* siRNA or scrambled siRNA, and treated with semaphorin-3A (250 ng/ml). Neurons were stained with β-tubulin III monoclonal antibodies. Control (A); Semaphorin-3A alone (B); *Nrp1* siRNA alone (C); D. *Nrp1* siRNA and semaphorin-3A (D); Scrambled siRNA alone (E); Scrambled siRNA and semaphorin-3A (F). Scale bar = 200 μm. (G,H, J) Recombinant Sema3a inhibits SGN outgrowth in whole cochlear cultures. (G, H) Three-dimensional confocal z-stacks from E17.5 cochlear cultures treated with 20nM of either control IgG-Fc or Sema3a-Fc. Tissue samples were stained with anti TUJ1 to mark SGNs (white) and Myo6 for HCs (blue). Note the dramatically diminished presence of fibers in the sample treated with Sema3a-Fc (arrowheads). Scale bar = 10um. (I) Average length of neurites grown from the SGN explants showing statistically significant decreased neurite outgrowth for Sama3a, *Nrp1* siRNA, and scrambled siRNA/Sema3a when compared to the control group. * p<0.001. (J) Panel J showing average percent neurite coverage of sensory epithelium. Error bars, SEM. * p<0.0001. (K) Western immunoblots of Nrp1 protein from SGN explants after transfection with either Nrp1 siRNA or scrambled siRNA control as indicated. β- Actin was used as a loading control.

To further investigate the role of Neuropilin-1/Sema-3A in mediating SGN migration and refinement, we used small interfering RNAs (siRNAs) targeting *Nrp1* mRNA to reduce neuropilin-1 protein levels in SGNs in cell culture. A transient transfection with predesigned siRNA oligonucleotides decreased neuropilin-1 protein expression as measured by Western immunoblotting (Fig 8). The SGN explant culture showed that semaphorin-3A, at a concentration of 250 ng/mL, mediated axonal repulsion (Fig 8B). The neurite outgrowth experiment was continued using the concentration of *Nrp1* siRNA oligonucleotide (50nM) that produced the greatest knockdown (approximately 60% decrease from the control). *Nrp1* knockdown caused by siRNA transfection decreased neurite outgrowth and abolished the ability of Sema3a to decrease neurite outgrowth, suggesting that Sema3a repulses SGNs in an Nrp1-depended manner (Fig 8I). In contrast, the negative control scrambled siRNA neither decreased Nrp1 protein nor abolished Sema3a activity. These experiments further support a key role for Nrp1/Sema3a signaling in cochlear innervation.

### Exogenous semaphorin-3A inhibits SGN outgrowth in cochlear explants

To investigate the interaction between semaphorin-3A and SGNs, we established whole cochlear cultures from E17.5 mice and placed them in media containing either control IgG-Fc or semaphorin-3A-Fc (20nM). To determine whether semaphorin-3A altered hair cell innervation, the tissue samples were labeled with TUJ1 and Myo6 antibodies and imaged by confocal microscopy. Compared to control samples that showed robust hair cell innervation (Fig 8G), samples treated with semaphorin-3A-Fc showed significantly reduced innervation of the sensory epithelium (Fig 8H). To quantify this change in innervation, high-resolution confocal z-stacks were taken from the volume of tissue occupied by the HCs. Compared to controls, semaphorin-3A decreased innervation around the sensory epithelium by nearly 60% (Fig 8J). These data suggest a possible role for semaphorin-3A in inhibiting SGN outgrowth.

### *Nrp1* conditional mutants show elevated hearing thresholds

For a detailed analysis of the entire auditory pathway in *Nrp1*^*fl/fl*^*;Pax2*^Cre^ mutants, we next evaluated OHC activity using DPOAE and neuronal responses by ABR wave I peak-to-peak amplitudes. DPOAEs, cochlear responses generated after two simultaneous pure tone frequencies, are objective indicators of OHC functional status [18]. OHC function was determined to be normal in *Nrp1* mutants as DPOAE levels for *Nrp1*^*fl/fl*^*;Pax2*^Cre^, *Nrp1*^*fl/+*^*;Pax2*^Cre^, and WT groups did not differ significantly at 2 and 4 months of age.

**Fig 9:**
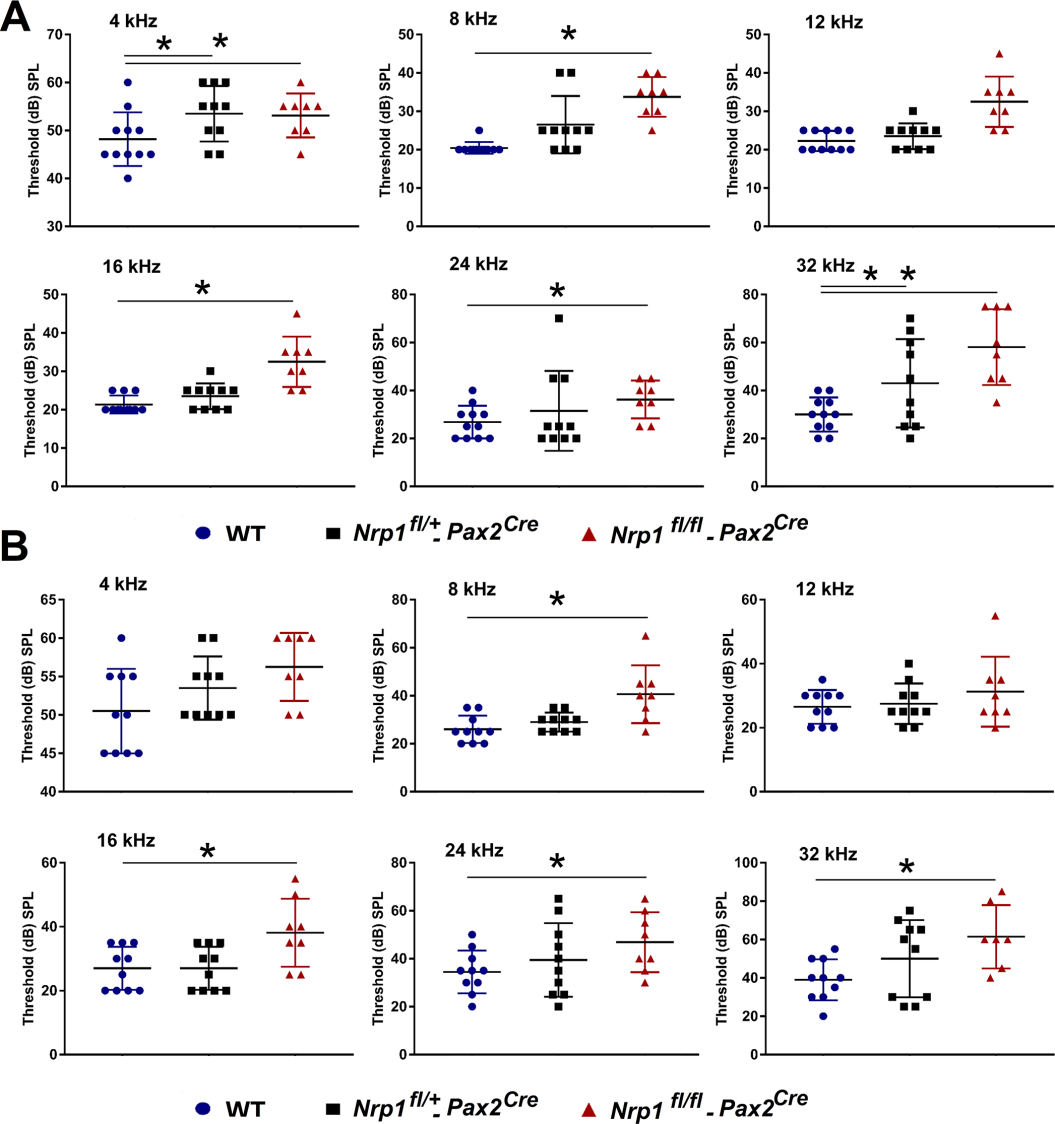
ABR for WT, *Nrp1*^*fl/+*^;*Pax2*^*Cre*^, and *Nrp1*^*fl/fl*^;*Pax2*^*Cre*^ mice at 2 and 4 months of age. ABR results of the 2-month-old mice (Panel A) showed that the hearing thresholds of the *Nrp1*^*fl/fl*^;*Pax2*^*Cre*^ group (n = 8) were significantly higher than WT controls (n = 11) at 4kHz, 8kHz, 16kHz, 24kHz, and 32kHz (A). At 4 months of age (Panel B), *Nrp1*^*fl/fl*^;*Pax2*^*Cre*^ mice (n = 8) showed elevated hearing thresholds at all tested frequencies except 12 kHz when compared to WT mice (n = 11) (B).

ABR test results of the 2-month-old mice showed that the hearing thresholds of the *Nrp1*^*fl/fl*^*;Pax2*^Cre^ group were significantly higher than WT controls at 4kHz, 8kHz, 16kHz, 24kHz, and 32kHz. At 4 months of age, *Nrp1*^*fl/fl*^*;Pax2*^Cre^ mice developed elevated hearing thresholds at all tested frequencies except 12 kHz when compared to WT controls (Fig 9).

**Fig 10:**
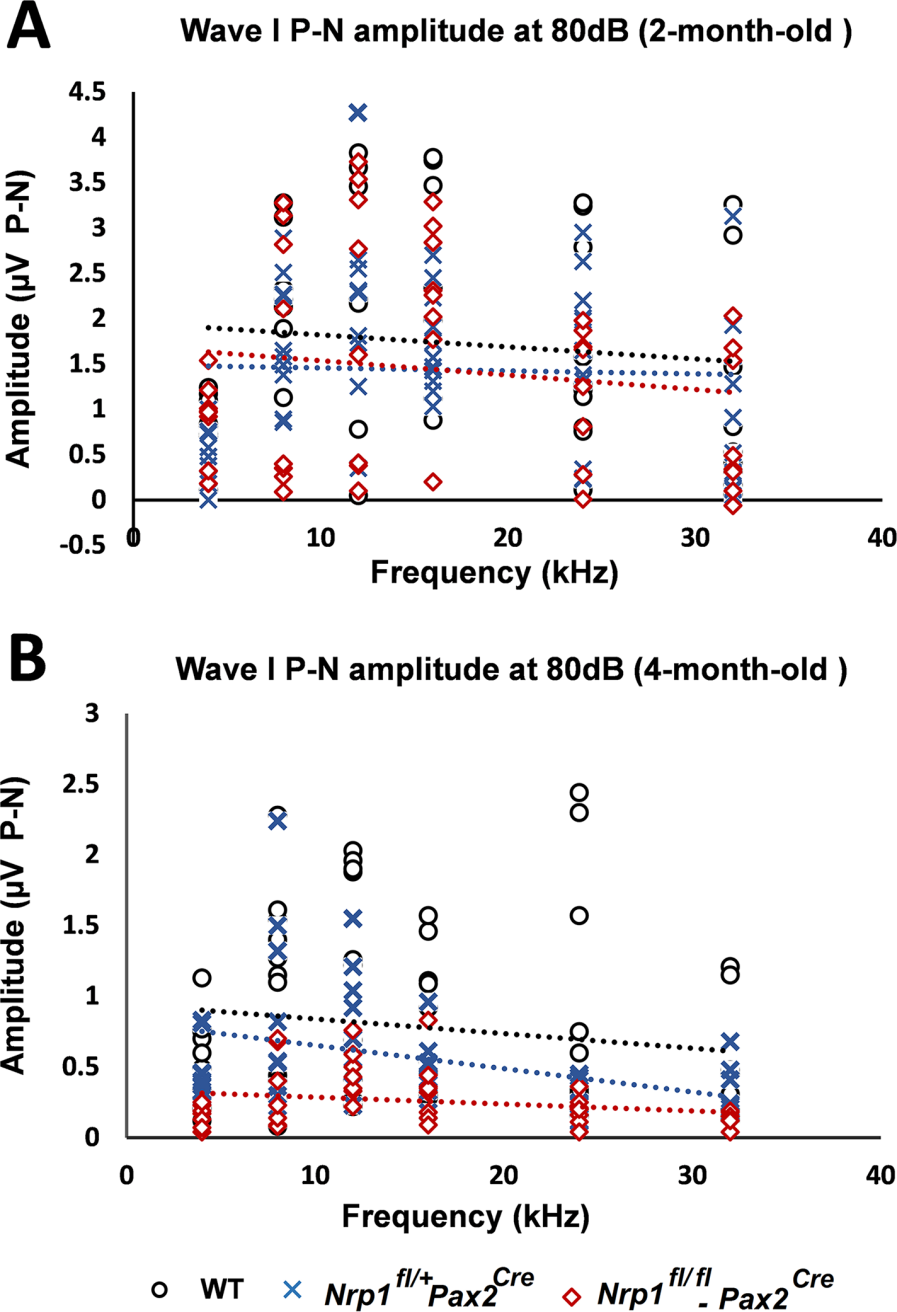
Wave I P-N amplitude for WT, *Nrp1*^*fl/+*^;*Pax2*^*Cre*^, and *Nrp1*^*fl/fl*^;*Pax2*^*Cre*^ mice at 2 and 4 months of age. Analysis of the wave I P-N amplitudes at 80dB suprathreshold did not show significant difference between *Nrp1*^*fl/+*^*Pax2*^*Cre*^ mice and WT controls at 2-month-old (A); however, statistically significant higher amplitudes at 8kHz, 12kHz, and 24kHz were shown in 4-month-old *Nrp1*^*fl/fl*^;*Pax2*^*Cre*^ mutants compared to controls (B). *p<0.05.

Peak-to-peak analysis of wave I was calculated from the ABR data described above. Wave I is thought to indicate the summed activity of SGN contact with hair cells, so a normal DPOAE with a diminished wave I peak would suggest dysfunction of the SGNs, IHCs, or the synapses between them [19]. At 2 months of age, no significant changes in wave I amplitude were found among the three groups of mice; however, the *Nrp1*^*fl/fl*^*;Pax2*^Cre^ mutants at 4 months of age recorded significantly lower wave I amplitudes than WT mice at 8 kHz, 12kHz, and 32 kHz. Paired with our immunostaining results of IHC defects in 4-month-old *Nrp1*^*fl/fl*^*;Pax2*^Cre^ mice, the reduced wave I amplitude suggests the contribution of cochlear neural damage in the hearing loss seen in 4-month-old *Nrp1* mutants (Fig 10).

### *Nrp1* mutants showed dilated microvessels in the stria vascularis

**Fig 11:**
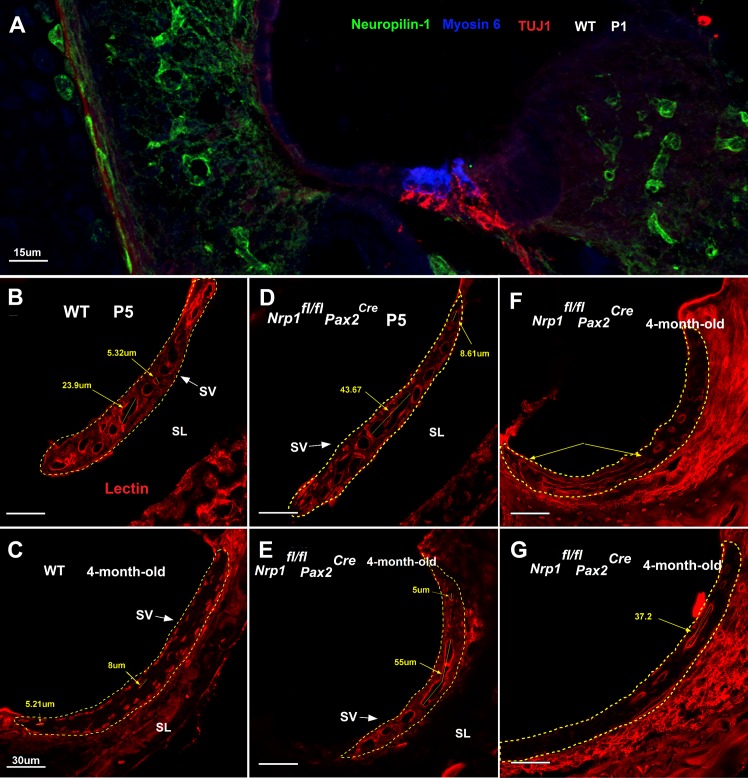
Stria vascularis abnormalities in *Nrp1*^*fl/fl*^;*Pax2*^*Cre*^ mutant mice at P5 and 4 months of age. Panel A showing neuropilin-1 expression (green) on vascular tissue of the cochlea. Arrows show neuropilin-1 stained vessels (A). Lectin immunostaining of WT and *Nrp1*^*fl/fl*^;*Pax2*^*Cre*^ cochleae at P5 and 4 months of age were performed to assess the morphology of the micro-vessels of the SV at the basal turn of the cochlea (n = 3 per group) (B-G). Grossly enlarged SV microvessels were apparent in both ages of *Nrp1*^*fl/fl*^;*Pax2*^*Cre*^ mutants. The minimum and maximum microvessel diameter of the *Nrp1*^*fl/fl*^;*Pax2*^*Cre*^ mice were 4.17μm and 43.67μm at P5, and 3.22 and 95μm in 4-month-old mice, respectively. The minimum and maximum microvessel diameters of the WT mice were 5.32μm and 23.90μm at P5, and 5.2μm and 26.15μm in 4-month-old mice, respectively. Panel B and C showing normal morphology of the microvessels of the WT mice at P5 and 4-month-old (B). *Nrp1*^*fl/fl*^;*Pax2*^*Cre*^ mutants at P5 showed some enlarged microvessels when compared to WT controls (D). Panel E-G showing vascular abnormalities of 3 different 4-month-old *Nrp1*^*fl/fl*^;*Pax2*^*Cre*^ mutants (E, G). Overall, the maximum microvessel diameter in 4-month-old *Nrp1*^*fl/fl*^;*Pax2*^*Cre*^ mice was 3.6 fold higher than WT controls. Scale bar = 30 μm.

The composition of endolymph and the maintenance of the endocochlear potential are determined by ion balance regulated by the stria vascularis (SV) [20]. We hypothesized that abnormal vascularization of the SV could lead to electrolyte imbalance, resulting in abnormal hearing thresholds. To test this hypothesis, we investigated the morphology of the micro-vessels of the SV at the basal turn of the cochlea (n = 3 per group). The lectin immunostaining of the *Nrp1*^*fl/fl*^*;Pax2*^Cre^ cochleae at P5 and 4-months-old demonstrated grossly enlarged SV microvessels (Fig 11). The minimum and maximum microvessel diameter of the *Nrp1*^*fl/fl*^*;Pax2*^Cre^ mice were 4.17μm and 43.67μm at P5, and 3.22μm and 95μm in 4-month-old mice, respectively. The minimum and maximum microvessel diameters of the WT mice were 5.32μm and 23.90μm at P5, and 5.2μm and 26.15μm in 4-month-old mice, respectively. Overall, the maximum microvessel diameter in 4-month-old *Nrp1*^*fl/fl*^*;Pax2*^Cre^ mice was 3.6 fold higher than WT controls. Thus, future endocochlear potential studies are needed to pinpoint the effect of *Nrp1* knockout on normal functioning of auditory hair cells.

## Discussion

There exists a growing literature supporting the notion that ARHL may be associated with degenerative changes in the cochlear nerve and its synapses [21, 22]. Although this phenomenon and that of the classically defined neural presbycusis are now well studied histologically, little is known about the molecular basis for this pathology. Using a meta-analysis GWAS approach we have defined several candidate regions for ARHL in mice, one of which included *Nrp1* [9]. *Nrp1* is a well-known factor in neuronal and cardiovascular development [13, 17]. Its homolog, *Nrp2* has been shown to be involved in inhibiting type I SGNs from the OHC region in the developing cochlea [10]. While substantial data exists for the role of *Nrp1* in tumorigenesis and embryonic development, to date, the role of *Nrp1* in the postnatal development of the cochlear apparatus remains unclear. According to previously published cochlear nerve microarray data, *Nrp1* expression in the spiral ganglion shows a peak at E16.5 followed by a dip between E16 and P0, and a general trend of increased expression up to two weeks into postnatal development [23]. These findings are consistent with our data as we found upregulated *Nrp1* postnatal expression in the organ of Corti and in the SGNs in the first postnatal week, a critical period for maturation of hair cell innervation which suggests a role for *Nrp1* in this process.

In the peripheral auditory system, the type I SGN afferent fibers undergo significant reorganization during embryonic development in mice [10]. In this study, we have identified *Nrp1* to be a critical component of this reorganization process. Previously, mice lacking normal *Nrp1* function (*Nrp1*^*Sema-*^ mutants) showed pathfinding defects in vestibular ganglion neurons [17], defasciculation of the intercostal nerves, and crossing bundles to neighboring nerves of the *Nrp1*^*Sema-*^ mutants [24]. Many previous studies have also demonstrated a role for semaphorin-3A in axonal chemorepulsion, including repulsion of sensory and cortical axons [11, 25]. Our SGN explant and semi-intact cochlear cultures also demonstrated semaphorin-3A repels SGNs, which suggests semaphorin-3A can normally inhibit SGN outgrowth. Complementary to these findings, *Nrp1*^*sema-/sema-*^ cochleae showed dramatically enhanced innervation by Syt-positive fibers during developmental stages, suggesting excessive innervation by SGNs possibly due to the absence of a repulsive signal. We do not yet know whether this was due to increased numbers of SGNs, increased complexity of individual SGNs, or ectopic innervation of the cochlea by a different population of neurons (e.g. vestibular neurons). During embryonic development, Type II SGNs pass by the IHCs and reach the OHC area then extend toward the base of the cochlea forming *en passant* contacts with 3 to 10 OHCs within the same row. These projections gather beneath the rows of OHCs to form 3 outer spiral bundles [15]. Here, we demonstrate that *Nrp1* conditional mutants show disorganized outer spiral bundles at early neonatal stages (P5) and that these pathfinding defects are apparent in the older (4-month-old) mice. In addition, *Nrp1*^*sema-/sema-*^ cochleae showed excessive numbers of SGNs present in the OHC region in late embryonic stages.

There were several pieces of evidence shown here that implicate *Nrp1* in age-related hearing loss. First, we found that SGN density was lost in the *Nrp1*^*fl/fl*^*;Pax2*^Cre^ cochleae over time (Fig 4). Since most of these neurons are likely type I SGNs, this potentially explains why the *Nrp1*^*fl/fl*^*;Pax2*^Cre^ mice showed elevated ABR thresholds and wave 1 amplitude reductions. Larger ABR wave I amplitude shifts at equal sound pressure levels are associated with greater auditory nerve threshold elevation [26]. Second, *Nrp1*^*fl/fl*^*;Pax2*^Cre^ cochleae showed conspicuous defects in the stria vascularis, which normally maintains the ionic composition of the endolymph and promotes normal auditory transmission. Although we did not detect any significant changes to otoacoustic emissions in the *Nrp1*^*fl/fl*^*;Pax2*^Cre^ mice (suggesting normal OHC function), it is possible that these mice have defects in IHC function that also, like the loss of SGNs, contributes to their altered hearing thresholds. The *Nrp1*^*fl/fl*^*;Pax2*^Cre^ mice did show reduced numbers of OHC ribbon synapses, but type II SGNs do not contribute to the canonical auditory pathway [27], so it is unlikely that this phenotypic defect contributes to the changes in hearing thresholds.

One curious finding here was that the *Nrp1*^*fl/fl*^*;Pax2*^Cre^ mice showed a profound loss of SGNs and only a mild loss of IHC ribbon synapses. Although overall the differences between controls were not statistically significant (Fig 3C), we did observe an almost 50% decrease in IHC ribbon synapses in 3 out of 5 of the 4-month-old *Nrp1* mutants examined. Since the majority of SGNs are type I and terminate on IHCs, it would be expected that there would be a commensurate reduction in IHC ribbon bodies. One obvious cause for this discrepancy is that many of the CtBP2-positive bodies in IHCs from the *Nrp1*^*fl/fl*^*;Pax2*^Cre^ cochleae may represent orphaned synapses that lack a postsynaptic terminal. A second less likely possibility is that some remaining type I SGNs extend collateral processes that form synapses with the IHCs.

Overall, these data suggest that reduced SGN density in 4-month-old *Nrp1* mutants, in addition to abnormal axonal pathfinding, lead to impairment of the OHCs synaptic integrity. Consistent with the abnormal neuronal phenotype in 4-month-old *Nrp1* deficient mice, we also observed a decline in ABR wave I amplitudes as they matured.

*Nrp2*, the other member of the neuropilin family, is responsible for encoding a transmembrane receptor protein with sequence homology to *Nrp1* but with different ligand binding affinities. Neuropilin-2 receptors bind Sema-3 subtypes 3C and 3F, VEGF-A and VEGF-C isoforms [28]. While *Nrp2* plays a role in neuronal pathfinding, it has not been linked to neuron survival [10]. We have shown, however, that *Nrp1* likely plays a role in long-term neuronal survival and maintenance throughout life as SGN cell counts diminish with age in *Nrp1* CKO mice. These results were consistent with previously published data showing that *Nrp1* is an essential factor in survival of the GnRH and trigeminal neurons by interacting with vascular endothelial growth factor (VEGF) ligands [29, 30].

While our results support a model in which *Nrp1* signaling is necessary for the establishment of SGN projections in the postnatal period, *Nrp1* also appeared to play an essential role in normal vascular development of the cochlea. Our results show that *Nrp1* deletion leads to enlarged vessels in the stria vascularis of early postnatal and adult mice, which may have an impact on the maintenance of the endocochlear potential [31].

## Methods

### Animals and genotyping

Animal procedures were performed at the Zilkha Neurogenetic Institute in accordance with the guidelines of the Institutional Care and Use Committee (IACUC) of the University of Southern California. *Nrp1*^*fl/fl*^ mice of mixed backgrounds (CBA/CaJ x C57BL/6J) strains were kindly provided by Dr. Henry Sucov. *Pax2*^Cre^ mice of mixed backgrounds strains (CBA/CaJ x C57BL/6J) were kindly provided by Dr. Takahiro Ohyama. In *Pax2*^Cre^ mice, Cre mRNA is detectable in the otic placode starting at the late presomite stage [32]. *Nrp1* CKO mice of either sex were obtained by crossing *Nrp1*^*fl/fl*^ mice to *Nrp1*^*fl/+*^;*Pax2*^*Cre*^ mice. For postnatal collections, P0 was defined as the day of birth. For genotyping of *Nrp1* knockout mice, polymerase chain reaction (PCR) was performed using the following primers: *Nrp1* forward 5’- AGGTTAGGCTTCAGGCCAAT-3’, *Nrp1* Reverse 5’ GGTACCCTGGGTTTTCGATT-3’; *Pax2*^Cre^ Forward 5’-GCCTGCATTACCGGTCGATGCAACGA-3’, *Pax2*^Cre^ Reverse 5’-GTGGCAGATGGCGCGGCAACACCATT-3’. The *Nrp1*^*sema-*^ line [17] was kindly provided by Dr. Alex Kolodkin of Johns Hopkins University. *Nrp1*^*sema-*^ mice were bred and maintained at either the Porter Neuroscience Research Facility (Bethesda, MD) under the guidelines of the NIDCD IACUC or at the Division of Comparative Medicine (Washington, DC) under the guidelines of the Georgetown University IACUC. *Nrp1*^*sema-*^ mice were maintained on a C57BL/6J background. Male and female heterozygous mice were bred to generate homozygous mutants and littermate controls. Genotyping was performed using the following WT and *Nrp1*^*sema-*^ specific primers: AGGCCAATCAAAGTCCTGAAA ACAGTCCC and AAACCCCCTCAATTGATGTTAACACAGCCC.

### Real-time PCR

Six-week-old C57BL/6 mice (n = 8) and DBA/2J mice (n = 7) were euthanized, and bilateral inner ears were harvested. Cochlear tissues were collected, and left and right ear samples were combined and immediately processed with RNAqueous Total RNA Isolation Kit (Life Technologies) according to manufacturer’s instructions. Total RNA was then converted to cDNA using the SuperScript III First-Strand Synthesis SuperMix (Life Technologies). PCR was performed using the primer pairs acquired from applied biosystems: assay ID: Mm00435379_m1. Each sample was run in triplicate along with the housekeeping gene, *GAPDH*. Relative quantities of the transcripts were determined using the 2−ΔΔCt method using *GAPDH* as a reference.

### Immunostaining of the cochlea

Cochlear whole mount sample preparation: Mouse cochleae were dissected after the second hearing measurement and were fixed with 4% PFA overnight. Fixed samples were decalcified using 10% EDTA, and dissected using the mouse cochlear dissection method from Eaton Peabody Laboratories at the Massachusetts Eye and Ear Institute website (http://www.masseyeandear.org/research/otolaryngology/investigators/laboratories/eaton-peabody-laboratories). For the *Nrp1*^*sema-*^ mice samples, embryonic cochleae were fixed for 30 minutes in 4% PFA and then rinsed extensively in 1X PBS before dissection and immunostaining.

Cochlear frozen section sample preparation: Fixed heads were sequentially dehydrated in 15% and 30% sucrose, embedded in Tissue-Tek O.C.T. compound (Sakura Finetek) and snap frozen on dry ice. Blocks were sectioned (12 μm thickness) on a Leica 3050 S cryostat in a cranial-to-caudal coronal direction.

SGN explant three-dimensional culture: Sensory epithelia of cochleae with attached SGNs were dissected from the E16.5 embryos and placed in Leibovitz’s L-15 medium (Invitrogen). The isolated SGNs were cut into four equal pieces starting from one turn away from the apex of cochlea. Each SGN explant was transferred in a drop of phenol red-free Matrigel (Corning) and placed on poly-D-lysine (50 μg/ml)-coated glass coverslips in a 24-well plate. After complete solidification of the Matrigel, DMEM/F12 medium supplemented with 10% fetal bovine serum, 1% N2 supplement, and 0.3 mg/ml ampicillin were added and maintained in the culture for 3 days. After fixation, dissected cochleae or tissue sections were permeabilized with 0.2% TrionX-100 followed by incubation in 10% blocking serum for 2 hours at room temperature. The samples were incubated with the primary antibody at 4°C for 24 to 48 hours and exposed to secondary antibodies for 2 hours at room temperature. Using a Carl Zeiss LSM 780 laser scanning microscope (AxioObserver.Z1), 3 representative images were taken for each slide, and the total, average, and maximal neurite lengths per SGN explant were measured using Metamorph software (Molecular Devices) [33, 34]. Antibodies used in this study were as follows: Alexa 488-conjugated mouse anti-neuron specific class III beta tubulin (anti-TUJ1) (1:300; Covance), rabbit anti-Neuropilin-1 (1:50;Abcam), rabbit anti-Semaphorin-3A (1:100; Abcam), mouse anti-CtBP2 (1:200; BD Biosciences), mouse-anti-TUJ1 (1:1,000, Covance), rabbit-anti-myosin VI (1:1,000, Proteus Biosciences), goat-anti-Sox2 (1:300, Santa Cruz Biotechnology), chicken-anti-synaptotagmin-1 (1:1,000, Aves Labs), mouse-anti-GAP43 (1:2,000, Chemicon), Alexa Fluor-488 anti-mouse (1:500; Life technologies), Alexa Fluor 594 anti-goat (1:500; ThermoFisher). Fluorescent dye Hoechst 33342 (0.1 μg/mL; Southern Biotech) was used for DNA labeling. Blocking peptide for Anti-Semaphorin-3A antibody (Abcam) was used for Sema-3A immunostaining (negative controls). For synaptic ribbon-to-hair cell ratios, tissue sections at the basal turn of the cochlea were selected, and the number of synaptic ribbons was compared separately to the number of inner hair cells and to the number of outer hair cells per section. The synaptic ribbon-to-hair cell ratios for WT and *Nrp1*^*fl/fl*^*;Pax2*^*Cre*^ 4-month-old mouse cochleae (n = 5 per group) and P5 mouse cochleae (n = 4 per group) were assessed. Spiral ganglion cells of the P5 and 4-month-old mice were counted at apical, mid, and basal turns of the cochlea (n = 5 per group at each turn). To quantify numbers of Syt1+ fibers in the OHC region of *Nrp1*^*sema-/sema-*^ cochleae and their littermate controls, we determined the number of fiber tracks extending into the OHC region and normalized that value to the longitudinal distance of the cochlea within that region. Sample sizes: 9 WT cochleae and 13 *Nrp1*^*sema-/sema-*^ cochleae.

### *In situ* hybridization

*In situ* hybridization was performed as previously described [35]. Briefly, embryonic day E15.5 heads were fixed in 4% paraformaldehyde in PBS overnight at 4°C, sunk in 30% sucrose in PBS at 4°C, incubated in Tissue-Tek O.C.T. compound (Sakura Finetek) at room temperature for 10 min and frozen on dry ice. Sections, 14μm thick, were cut using a Leica 3050 S cryostat. RNA probes for mouse *Nrp1* (GE Dharmacon, Clone ID 6409596) and mouse *Sema3a* (GE Dharmacon, Clone ID 30532393) were synthesized, labeled with digoxigenin, and hydrolyzed by standard procedures. *In situ* hybridization images were obtained under bright-field microscopy (BZ9000; Keyence, Osaka, Japan).

### *Nrp1* siRNA knockdown, Western immunoblotting

SGN explants in antibiotic-free medium were transfected with 50 nM predesigned *Nrp1* siRNA oligonucleotides (Santa Cruz Biotechnology) using Lipofectamine 3000 (Invitrogen) as per manufacturer’s instructions. Some explants were treated with 250 ng/ml Sema-3A-Fc (R&D Systems). We used a scrambled siRNA oligonucleotide that did not exhibit homology to any known encoding region as a negative control. The siRNA-mediated knockdown efficiency was determined by Western immunoblotting. After *Nrp1* siRNA transfection and semaphorin-3A treatments, the Matrigels covering SGN explants were removed with cell recovery solution (Corning) and the explants were homogenized in RIPA lysis buffer supplemented with a cocktail of protease inhibitors (Santa Cruz Biotechnology). Explant extracts were electrophoresed on 7.5% SDS-polyacrylamide gels and the separated proteins were transferred to a polyvinylidene fluoride membrane. The membranes were blocked with 5% dry milk and then incubated with rabbit-anti-Neuropilin-1 (1: 1000 dilution; Abcam) overnight, followed by incubation with a goat anti-rabbit secondary antibody conjugated with horseradish peroxidase. The immunoreactive proteins were visualized by the Supersignal West Femto Maximum Sensitivity Substrate kit (Thermo Scientific). The bands were quantitated by densitometric scanning using the Omega 12 IC Molecular Imaging System and the Ultra Quant software. β-Actin loading control was carried out.

### Cochlear explant experiments and quantification procedures

E17.5 cochleae were dissected in chilled HBBS/HEPES, and the cochlear capsule and stria vascularis were removed. The tissue samples were placed on polycarbonate filters and incubated at 37°C. The culture medium: DMEM supplemented with 10% fetal bovine serum, 0.2% N2 (Thermo-Fisher Scientific), and 0.0001 ciprofloxacin (Sigma). Either purified Sema-3A-Fc (R&D Systems) or human IgG-Fc (Jackson Immunoresearch) was added to the medium for a final concentration of 20nM. After 20hr of incubation, the cochleae were removed from the filters, fixed for 15 min in cold 4% PFA, then processed for immunostaining (anti-TUJ1 and anti-Myo6) and confocal imaging. To image the tissue samples, confocal z-stacks were acquired starting at the “top” of the hair cells (lumen of the cochlear duct) through the “bottom” of the supporting cells (past the basement membrane). To quantify how Sema-3A-Fc altered cochlear innervation, maximum-intensity z-projects were generated only from the z-planes that included the hair cells. Using ImageJ, a region of interest (ROI) around the inner and outer hair cells was selected from each sample and the area occupied by TUJ1-positive neurons was normalized to the area of the ROI. For each sample, equivalent thresholding was used.

### Auditory Brainstem Response (ABR) and Distortion Product Otoacoustic Emissions (DPOAEs)

ABR and DPOAE testing was performed for *Nrp1*^*fl/fl*^*;Pax2*^*Cre*^ (n = 8), *Nrp1*^*fl/+*^*;Pax2*^*Cre*^ (n = 8), and WT mice (n = 11) at 2 and 4 months of age. DPOAE testing was performed for *Nrp1*^*fl/fl*^*;Pax2*^*Cre*^ and WT mice at 4 months of age. Stainless-steel electrodes were placed subcutaneously at the vertex of the head and the right mastoid with a ground electrode at the base of the tail. Auditory signals were presented as tone pips with a rise and fall time of 0.5 msec and a total duration of 5 msec at the frequencies 4, 8, 12, 16, 24, and 32 kHz. Tone pips were delivered below threshold and then increased in 5 dB increments until the goal of 100 dB was reached. Signals were presented at a rate of 30/second. Responses were filtered with a 0.3 to 3 kHz pass-band (x10000 times). For each stimulus intensity, 512 waveforms were averaged. Hearing threshold was determined by inspection of ABR waveforms and was defined as the greatest intensity at which no reliable ABR waveforms were generated. Data were stored for offline analysis of peak-to-peak (P1-N1) values for wave 1 amplitudes. ABR thresholds were determined by an independent observer who was blinded to the genotypes of the mice. Peak-to-peak (P1-N1) values for wave 1 amplitudes were calculated using the ABR Peak Analysis software (Bradley Buran, Eaton-Peabody Laboratory). Distortion product otoacoustic emissions (DPOAEs) were recorded with a custom-designed transducer and data-acquisition system based on a National Instruments PXI hardware configuration developed at the Eaton-Peabody Laboratories, Massachusetts Eye and Ear Infirmary. The 2f1-f2 DPOAE was measured with an f2/f1 ratio of 1.22 for f2 = 8, 11, 16, 22 and 32 kHz at primary tone levels (L2) presented from 20 to 70 dB SPL in 10 dB steps to form an input/output function. DPOAE threshold was determined from the input/output function as the extrapolated primary tone level (L2) required to produce a DPOAE of 0 dB SPL. DPOAEs with at least 3 dB signal-to-noise (SNR) ratio were included in all analyses; however, when the SNR was < 3 dB, the DPOAE was assigned the level of the noise floor and included. This was done to avoid excluding the very lowest level DPOAEs, which carry information by virtue of their reduced amplitude. DP-grams (DPOAE amplitude across frequency, 8–32 kHz) were also constructed at L2 = 70 dB SPL for all mice; only points > 3 dB SNR were included in this analysis.

### Statistical analysis

Statistical analysis for ABR, and wave I amplitude were performed using analysis of variance (one-way ANOVA) and post hoc comparisons with Fisher’s LSD test. For neurite outgrowth analysis, Kruskal-Wallis Test, and Dunn's Test were used. Synaptic ribbon count, and spiral ganglion neuron counts were performed using the student’s t-test. GraphPad Prism 7 software was used to perform the tests. Continuous variables with normal distribution were expressed as mean +/- standard deviation (SD). A 2-tailed p value less than 0.05 indicated statistically significant differences.

## Conclusions

The results of this study support a potential role for *Nrp1* expression variation to be associated with ARHL in mice. Furthermore, our findings suggest *Nrp1* plays a role in the postnatal development and maintenance of the cochlear neuronal network. Our findings also support the role of *Nrp1* in normal development of the stria vascularis microvasculature. Taken together, these data suggest that both neuronal and vascular abnormalities of *Nrp1* deficient mice contribute to abnormal hearing in 4-month-old mice. To further understand the impact of Neuropilin-1/Sema-3A on ARHL, future studies will likely include investigation of tissue-specific knock outs of *Sema3a* and *VEGF-B* in the inner ear, neuronal cell type-specific immunostaining of these selected animal models, and assessment of cochlear hemodynamics and electrophysiology of the *Nrp1* deficient mice.

## Supporting information

S1 Fig *Nrp1* expression variation in the cochlear tissue of C57BL/6J and DBA/2J mice. Quantitative real-time PCR revealed 1.78-fold higher *Nrp1* expression in adult (6-week-old) DBA/2J mice (1.96) as compared to C57BL/6J (1.09). *p<0.01.
(TIF)

S1 Dataset Data underlying Fig 3. Presynaptic marker CtBP2 counts of the IHCs and OHCs.
(CSV)

S2 Dataset Data underlying Fig 4. SGN density in 4-month-old WT and Nrp1flfl;Pax2Cre mutant mice.
(CSV)

S3 Dataset Data underlying Fig 7O. Syt+ SGN processes projecting into the OHC region.
(CSV)

S4 Dataset Data underlying Fig 8I. The average length of neurite outgrowth.
(CSV)

S5 Dataset Data underlying Fig 8J. The average percent neurite coverage of sensory epithelium.
(CSV)

S6 Dataset Data underlying Fig 9. Auditory Brainstem Response.
(CSV)

S7 Dataset Data underlying Fig 10. ABR Wave I amplitudes.
(CSV)

S8 Dataset Data underlying S1 Fig. Nrp1 expression in the cochlear tissue of C57BL6J and DBA2J mice.
(CSV)
