# Supplementary figures and images for: Role of Neuropilin-1/Semaphorin-3A signaling in the functional and morphological integrity of the cochlea
Source: PLoS Genet. 2017 Oct 23;13(10):e1007048. doi: 10.1371/journal.pgen.1007048 (PMC5695633; doi:10.1371/journal.pgen.1007048)

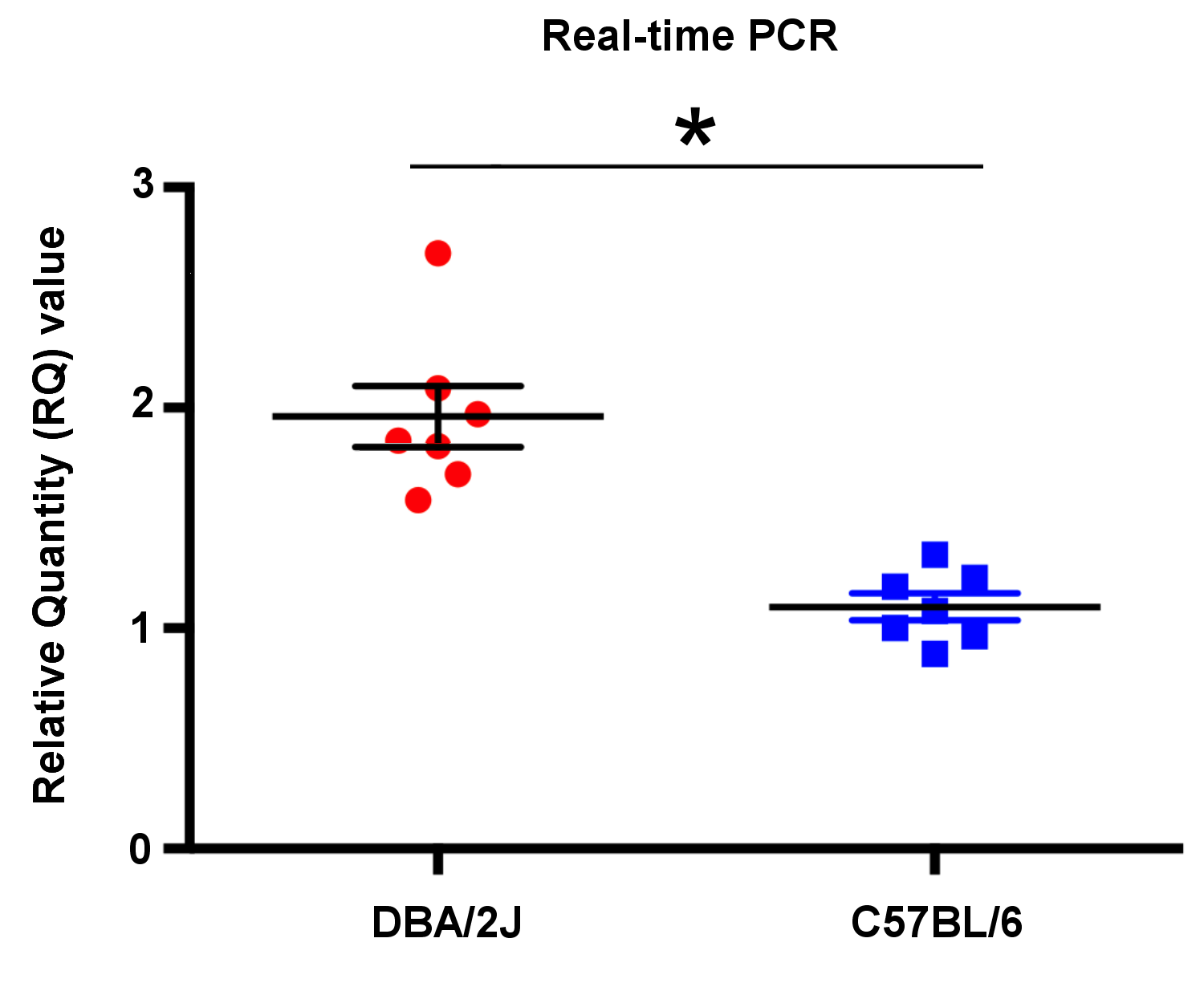

Supplement: S1 Fig — Quantitative real-time PCR revealed 1.78-fold higher Nrp1 expression in adult (6-week-old) DBA/2J mice (1.96) as compared to C57BL/6J (1.09). *p<0.01. (TIF) [file pgen.1007048.s001.tif]
